# Comparisons of host mitochondrial, nuclear and endosymbiont bacterial genes reveal cryptic fig wasp species and the effects of *Wolbachia *on host mtDNA evolution and diversity

**DOI:** 10.1186/1471-2148-11-86

**Published:** 2011-04-01

**Authors:** Xiao-Jing Sun, Jin-Hua Xiao, James M Cook, Gui Feng, Da-Wei Huang

**Affiliations:** 1 Key laboratory of Zoology Systematics and Evolution, Institute of Zoology, Chinese Academy of Sciences, Beijing, 100101, PR China; 2 Graduate School of the Chinese Academy of Sciences, Beijing, 100039, PR China; 3 School of Biological Sciences, University of Reading, Reading, Berkshire, RG6 6BX, UK; 4 College of Life Sciences, Hebei University, Baoding, 071002, PR China

## Abstract

**Background:**

Figs and fig-pollinating wasp species usually display a highly specific one-to-one association. However, more and more studies have revealed that the "one-to-one" rule has been broken. Co-pollinators have been reported, but we do not yet know how they evolve. They may evolve from insect speciation induced or facilitated by *Wolbachia *which can manipulate host reproduction and induce reproductive isolation. In addition, *Wolbachia *can affect host mitochondrial DNA evolution, because of the linkage between *Wolbachia *and associated mitochondrial haplotypes, and thus confound host phylogeny based on mtDNA. Previous research has shown that fig wasps have the highest incidence of *Wolbachia *infection in all insect taxa, and *Wolbachia *may have great influence on fig wasp biology. Therefore, we look forward to understanding the influence of *Wolbachia *on mitochondrial DNA evolution and speciation in fig wasps.

**Results:**

We surveyed 76 pollinator wasp specimens from nine *Ficus microcarpa *trees each growing at a different location in Hainan and Fujian Provinces, China. We found that all wasps were morphologically identified as *Eupristina verticillata*, but diverged into three clades with 4.22-5.28% mtDNA divergence and 2.29-20.72% nuclear gene divergence. We also found very strong concordance between *E. verticillata *clades and *Wolbachia *infection status, and the predicted effects of *Wolbachia *on both mtDNA diversity and evolution by decreasing mitochondrial haplotypes.

**Conclusions:**

Our study reveals that the pollinating wasp *E. verticillata *on *F. microcarpa *has diverged into three cryptic species, and *Wolbachia *may have a role in this divergence. The results also indicate that *Wolbachia *strains infecting *E. verticillata *have likely resulted in selective sweeps on host mitochondrial DNA.

## Background

The system of figs and fig wasps is considered to be a classic example of coevolved mutualism. It is well known that in general each fig species has a unique pollinator, which is called the "one-to-one" rule. However, more and more examples of co-pollinators (two or more pollinating wasp species on a fig) have broken the "one-to-one" rule [1-10]. However, we do not yet know how they evolve. They might be distantly related species, suggesting host shifts, or sister species, suggesting speciation on the current host [11,12]. Host shifts might be more likely when a fig colonises a new habitat, or is near the edge of its geographic range, because the normal pollinator is rare or absent. In this scenario, the co-pollinators are usually not closely related species [2,5,8]. Alternatively, sister co-pollinators may evolve from a recent speciation event in the pollinator that is not accompanied by fig radiation [3,11,12]. However, the exact mode of speciation for co-pollinators has not been well understood yet.

*Wolbachia *bacteria are the most common intracellular bacteria in arthropods and nematodes, and can manipulate host reproduction in many ways [13]. Cytoplasmic incompatibility (CI), the most common effect on host reproduction, usually occurs between infected males and uninfected females (or females infected by a different incompatible *Wolbachia *strain), inducing progeny sterility or mortality [13]. This post-zygotic reproductive isolation can potentially cause or facilitate host speciation [14-18]. Fig wasp species have a very high incidence of *Wolbachia *infection [19-21], and previous work suggests that *Wolbachia *might have an influence on fig wasp speciation, because cryptic pollinator species have different *Wolbachia *infections [3]. So, *Wolbachia *may play a potential important role in co-pollinator speciation.

In addition, *Wolbachia *can influence host mitochondrial DNA evolution. Because *Wolbachia *and mitochondria are co-transmitted maternally, the spread of *Wolbachia *can result in the hitchhiking of mitochondrial haplotypes. One particular mitochondrial haplotype can sweep through a population, via hitchhiking, associated with the sweep of *Wolbachia *[22,23]. This affects mtDNA evolution by decreasing mitochondrial haplotype diversity and also sometimes the mtDNA divergence between species [15,22-27], and thus can confound phylogenies and barcodes based on host mtDNA [28,29]. Accordingly, we look forward to understanding the effect of *Wolbachia *on mtDNA evolution of fig wasps.

*Ficus microcarpa *Linn. is a functionally monoecious fig species. It is pollinated solely by *Eupristina verticillata *Waterston [30]. Here, we investigate the genetic variance and *Wolbachia *infection status of *Eupristina verticillata *in order to explore whether co-pollinators exist, the association between *Wolbachia *infection and co-pollinators speciation, and the effect of *Wolbachia *on host mtDNA evolution.

## Results

### Morphological study

We extracted DNA from the specimens non-destructively, so the specimens can be conserved as vouchers for morphological study. According to the keys of Bouček and Wiebes [30,31], all the specimens are morphologically identified as *Eupristina verticillata*.

### *Wolbachia *infection

After initial screening for *Wolbachia *infection, we selected 76 pollinator specimens (35 infected, 41 uninfected) for further research. Only one specimen is infected by two *Wolbachia *strains, while the other 34 specimens are infected by a single strain (Table 1).

**Table 1:** Summary of *E. verticillata *samples and their genetic characteristics.

Locations	Wasp code	*COI *clades	ITS2 clades	*Wolbachia * strains*
Haikou, Hainan	HK-EV-20	1	Ⅰ	wEv1
	HK-EV-22	1	Ⅰ	wEv1
	HK-EV-27	1	Ⅰ	wEv1
	HK-EV-29	1	Ⅰ	wEv1
	HK-EV-30	1	Ⅰ	wEv1
	HK-EV-3	1	Ⅰ	-
	HK-EV-7	1	Ⅰ	-
	HK-EV-10	1	Ⅰ	-
	HK-EV-16	1	Ⅰ	-
	HK-EV-18	1	Ⅰ	-

Danzhou, Hainan	DZ-EV-1	2	Ⅱ	wEv2
	DZ-EV-2	2	Ⅱ	wEv2
	DZ-EV-3	2	Ⅱ	wEv2
	DZ-EV-4	2	Ⅱ	wEv2
	DZ-EV-5	2	Ⅱ	wEv2
	DZ-EV-6	2	Ⅱ	-
	DZ-EV-7	2	Ⅱ	-
	DZ-EV-8	2	Ⅱ	-
	DZ-EV-9	2	Ⅱ	-
	DZ-EV-10	2	Ⅱ	-

Xidao, Hainan	XD-EV-2	2	Ⅱ	wEv2
	XD-EV-5	1	I	wEv1
	XD-EV-7	2	Ⅱ	wEv2
	XD-EV-10	2	Ⅱ	wEv2
	XD-EV-13	2	Ⅱ	wEv2
	XD-EV-3	2	Ⅱ	-
	XD-EV-6	2	Ⅱ	-
	XD-EV-8	2	Ⅱ	-
	XD-EV-9	2	Ⅱ	-
	XD-EV-12	2	Ⅱ	-

Sanya, Hainan	SY-EV-22	1	I	wEv1
	SY-EV-23	1	I	wEv1
	SY-EV-24	1	I	wEv1
	SY-EV-26	1	I	wEv1
	SY-EV-28	1	I	wEv1
	SY-EV-15	1	I	-
	SY-EV-19	1	I	-

Lingshui, Hainan	LS-EV-16	1	I	wEv1
	LS-EV-17	2	II	wEv2
	LS-EV-18	1	I	wEv1
	LS-EV-19	1	I	wEv1
	LS-EV-20	2	II	wEv2
	LS-EV-12	1	I	-
	LS-EV-14	2	II	-
	LS-EV-21	2	II	-
	LS-EV-26	2	II	-
	LS-EV-28	2	II	-

Wanning, Hainan	WN-EV-1	1	I	wEv1
	WN-EV-5	1	I	wEv3
	WN-EV-7	1	I	wEv1
	WN-EV-8	1	I	wEv1
	WN-EV-9	1	I	wEv1
	WN-EV-2	1	I	-
	WN-EV-3	1	I	-
	WN-EV-4	1	I	-
	WN-EV-11	1	I	-

Baisha, Hainan	BS-EV-4	2	II	wEv2
	BS-EV-14	2	II	wEv2
	BS-EV-16	2	II	wEv2 wEv3
	BS-EV-17	2	II	wEv2
	BS-EV-19	2	II	wEv2
	BS-EV-5	2	II	-
	BS-EV-8	2	II	-
	BS-EV-10	1	I	-
	BS-EV-15	2	II	-
	BS-EV-18	2	II	-

Wenchang, Hainan	WC-EV-21	3	Ⅲ	-
	WC-EV-22	3	Ⅲ	-
	WC-EV-23	3	Ⅲ	-
	WC-EV-24	3	Ⅲ	-
	WC-EV-25	3	Ⅲ	-

Wuping, Fujian	FW-EV-1	1	Ⅰ	-
	FW-EV-2	1	Ⅰ	-
	FW-EV-3	1	Ⅰ	-
	FW-EV-5	1	Ⅰ	-
	FW-EV-9	1	Ⅰ	-

* A line means no *Wolbachia*.

**Figure 1:**
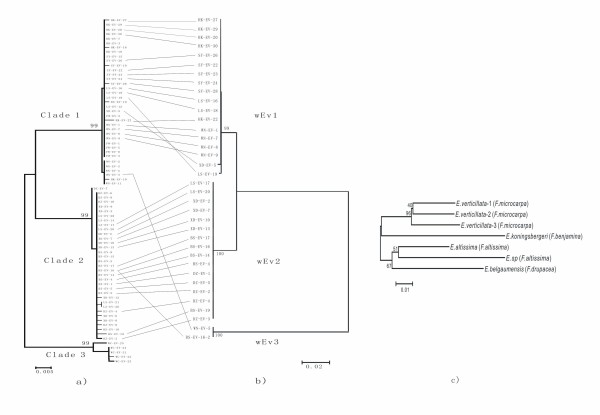
**Phylogeny of *Eupristina *species and Correlated divergences of *E. verticillata *and *Wolbachia *strains**. a) the tree based on *E. verticillata COI *gene. b) the tree based on *Wolbachia wsp *gene. c) phylogeny of *Eupristina *species based on *COI *gene.

In view of the NJ-tree based on *wsp *sequences and the divergence among *wsp *haplotypes (Figure 1b), we recognise three *Wolbachia *strains (wEv1, wEv2 and wEv3). The divergence between wEv1 and wEv2 is much smaller (2.96%) than that between wEv1 and wEv3 (22.44%), and between wEv2 and wEv3 (23.20%).We then searched for similar *wsp *sequences on the NCBI database. The results show that wEv1 and wEv2 are 97-100% similar to the *wsp *sequences found in *E. verticillata *in Australia-Asia [20,21], whereas wEv3 is similar to a great number of *wsp *sequences found in many other insects, including other fig wasps, gall wasps, mosquitoes, bugs, fruit flies and so on.

### Phylogeny and molecular variance

We obtain *COI *and ITS2 sequences for all 76 pollinator specimens. There are 24 unique *COI *haplotypes of 652bp in length, of which 68 nucleotide sites are polymorphic and 47 are parsimony informative. The NJ-tree based on *COI *sequences (Figure 1a) shows that all the specimens cluster into 3 distinct clades with high bootstrap values. We name them as Clade 1, Clade 2 and Clade 3. The mean *COI *genetic divergence within each clade is tiny (0.11%, 0.11%, 0.49% respectively), while the mean divergence among the clades is large (4.22%-5.28%) enough to suggest different species.

We also obtain 76 ITS2 sequences, whose length vary from 522 bp to 545 bp. The NJ-tree based on ITS2 sequences (Additional file 1) also shows three distinct clades (Clade I, Clade II and Clade III). The sequences within each clade are nearly or absolutely identical. The divergence among the clades, due mostly to length variance, is 2.29% between Clade I and II, 20.28% between Clade II and III, and 20.72% between Clade I and III. Remarkably, the NJ-trees based on *COI *and ITS2 sequences have very similar topologies, and the members which cluster together in *COI *clades also cluster together in ITS2 clades (Additional file 1).

### Distribution of pollinators

A summary of *E. verticillata *samples and their genetic characteristics is shown in Table 1. All samples from Haikou, Sanya, Wanning, Wuping fall into Clade 1 and those from Danzhou and Wenchang belong to Clade 2 and Clade 3, respectively. For Xidao, Lingshui and Baisha, individuals of Clade 1 and Clade 2 coexist.

### Mitochondrial DNA polymorphism and evolution pattern

We mapped the status of *Wolbachia *infection onto the *E. verticillata *clades based on *COI *sequences. This reveals very strong concordance between *E. verticillata *clades and *Wolbachia *infection status. Clade 1 and Clade 2 mainly harbour wEv1 and wEv2, respectively, while Clade 3 specimens are free from *Wolbachia *infection (Table 1). In order to examine the effect of *Wolbachia *on host mitochondrial DNA, we compared patterns of mitochondrial DNA polymorphism and evolution among the clades. The results are shown in Table 2.

**Table 2:** Comparison of *COI *gene evolution of *E. verticillata *samples clades.

	n	h	Hd	S	Pi	D	Tajima's D	Fu and Li's D	Fu and Li's F
Clade 1	37	9	0.535	9	0.00109	0.11%	-1.98912*	-3.55505**	-3.59153**
Clade 2	34	11	0.547	11	0.00108	0.11%	-2.31606***	-3.75589**	-3.87463**
Clade 3	5	4	0.900	8	0.00491	0.49%	-1.17432	-1.17432	-1.22979

n = no. of fig wasps samples; h = no. of mitochondrial DNA haplotype; Hd = haplotype diversity; S = no. of segregating sites; Pi = nucleotide diversity; D = genetic divergence. *: p < 0.05;**: p < 0.02;***: p < 0.01.

The mean genetic divergence (D), haplotype diversity (Hd) and nucleotide diversity (Pi) among the three clades are significantly different. They are all much lower in Clade 1 and 2 than in Clade 3. In addition, we could reject neutral evolution for Clade 1 and 2 with neutrality values (Tajima's D, Fu and Li's D and Fu and Li's F) significantly less than zero, but not for Clade 3. In summary, we detect predicted effects of *Wolbachia *infection on both mtDNA diversity and evolution.

## Discussion

### Cryptic co-pollinators

In this research, data from both mitochondrial (*COI*) and nuclear (ITS2) markers indicate that *E. verticillata *wasps fall into three distinct species (clades). *COI *divergence among these clades is large (4.22%-5.28%) enough to suggest that these are different species. *COI *is the gene used for animal DNA barcoding and has the potential to facilitate both the identification of known species and the discovery of new ones [32]. Hebert et al. (2003) studied the ability of *COI *to distinguish 2238 species across 11 animal phyla, and the result revealed that more than 98% of species pairs showed greater than 2% sequence divergence [32]. In this study, the *COI *divergence is greater than 2%, so it suggests the existence of cryptic species within the morphologically defined *E. verticillata*. However, because *Wolbachia *can confound DNA barcoding based on mtDNA, we need nuclear markers to support the conclusion from the analysis of mtDNA. Nuclear marker ITS2 sequences, which are not affected by *Wolbachia*, also support the conclusion of cryptic species. ITS2 has been studied in other hymenopterans, and this nuclear DNA fragment shows high conservation within species and high divergence between congeneric species [33]. In our study, ITS2 sequences within each clade are nearly identical but diverge considerably among clades (2.29%, 20.28% and 20.72%). Therefore, according to the tree topologies and the divergence of *COI *and ITS2, we define the specimens in Clade 1, 2 and 3 as *E. verticillata*-1, *E. verticillata*-2 and *E. verticillata-*3.

### How can cryptic co-pollinators occur?

As background mentioned, co-pollinators may occur as a result of host shift or speciation. In South China, *F. microcarpa *is widespread, so host shift event seems unlikely. In addition, survey of fig wasps in Hainan and other provinces in South China does not reveal that *E. verticillata *exists on other host figs (unpublished data). All the above information suggests that occurrence of co-pollinators on *F. microcarpa *is unlikely to be due to host shift.

Alternatively, co-pollinators may evolve from a recent speciation event in the pollinator that is not accompanied by fig radiation [3,11,12]. In this circumstance, the co-pollinators are likely to be sister or close related species [3,11,12]. Phylogenetic analysis indicates that the three species we found are not only cryptic species, but also closely related species (analysis combined with other *Eupristina *species, Figure 1c). Based on the commonly used mitochondrial DNA clock rate of 2.3% pairwise divergence/Myr [34], *E. verticillata*-1 diverged from *E. verticillata*-2 about 1.8 million years ago, and *E. verticillata*-3 diverged from *E. verticillata*-1 and *E. verticillata*-2 about 2.3 million years ago. Although *E. verticillata*-3 seems distant from the other two species particularly based on ITS2 sequences, in view of the phylogeny and identical morphology, we still expect that the three species share the same ancestor. Together with the evidence that in some locations (Xidao, Lingshui and Baisha), *E. verticillata*-1 and *E. verticillata*-2 occur on the same tree, we conclude that *E. verticillata *has diverged into three species on the same host. This pattern might be attributed to the comparatively longer generation time of figs compared to that of wasps [4,11].

New species often arise via geographic isolation, but the current geographic distributions of the three species do not suggest geographic isolation (Additional file 2). In some locations, *E. verticillata*-1 and *E. verticillata*-2 coexist. *E. verticillata*-3 is only found in Wenchang, but this phenomenon might only reflect low sampling to date. Additionally, speciation may also be driven by *Wolbachia*-induced reproductive incompatibility. Previous studies have indicated that *Wolbachia *can induce reproductive incompatibility, and then facilitate host speciation [14-18]. In this study, we note that the three species have different *Wolbachia *infection status. *E. verticillata*-1 and *E. verticillata*-2 are infected by closely related *Wolbachia *strains, wEv1 and wEv2, while *E. verticillata*-3 is uninfected. A blast search for similar *wsp *sequences on the NCBI database indicates that wEv1 and wEv2 are specifically related to *E. verticillata*. The strong correlation between phylogenetic trees of wasp species and *Wolbachia *strains suggests that *Wolbachia *may be involved in the divergence and speciation of hosts. In addition, we calculated the time when wEv1 and wEv2 diverged based on the *wsp *divergence rate (0.2% pairwise divergence/Myr) [35]. The divergence between wEv1 and wEv2 occurred about 15 MYA, earlier than the host wasp divergence. This makes it possible that wEv1 and wEv2 play a role in host speciation. Although our results show that wEv1 and wEv2 may be involved in host speciation, we can not demonstrate that *Wolbachia *cause reproductive isolation among these three host species. So, the exact mode of speciation for three *E. verticillata *still needs to be explored.

### Effect of *Wolbachia *on host mitochondrial DNA polymorphism and evolution pattern

Many explanations have been proposed for lower mitochondrial DNA polymorphism and deviation from neutral evolution, including population expansion from a bottleneck [36,37], selective sweep [26] and selection against weakly deleterious mutations [38]. We analyse the population size change by DnaSP, and get no evidence for population expansion (Additional file 3) [39]. Therefore, we should consider the other two possibilities. It is notable that *E. verticillata*-1 and *E. verticillata*-2 are infected by *Wolbachia *and show lower mitochondrial DNA polymorphism than uninfected *E. verticillata*-3. The perfect concordance of mitochondrial DNA polymorphism and *Wolbachia *infection status suggests that *Wolbachia*-associated selective sweeps of the mitochondrion have occurred in *E. verticillata*-1 and *E. verticillata*-2. Decreased mitochondrial DNA polymorphism as a consequence of *Wolbachia *infection has also been reported in several other insects [15,22-27]. We also find that the *COI *genes of *E. verticillata*-1 and *E. verticillata*-2 deviate significantly from neutral evolution while this is not so for *E. verticillata*-3. Therefore, this is also likely due to an mtDNA sweep associated with the spread of *Wolbachia*, making the mtDNA undergo purifying selection. In addition, a selection against the weakly deleterious mutation may also induce lower mitochondrial DNA polymorphism and deviation from neutral evolution, and it needs to be explored further.

## Conclusions

Our study reveals the molecular variance within *E. verticillata*, and indicates the presence of cryptic species. Further analyses suggest that the occurrence of cryptic species is attributable to successive speciation. A survey on the *Wolbachia *infection in *E. verticillata *points to a strong correlation between *Wolbachia *strains and the pollinator species, which may indicate an important role of *Wolbachia *in the formation of the cryptic species. Our study also reveals that *Wolbachia *strains infecting *E. verticillata *have likely resulted in selective sweeps on host mitochondrial DNA.

## Methods

### Specimen collection and identification

Ripe syconia were collected from nine *F. microcarpa *trees each growing at a different location in Hainan and Fujian provinces, China, between 2006 and 2008 (Table 1). Wasps from more than 200 syconia per tree were allowed to emerge and then stored in 95% ethanol at -20℃. All pollinator specimens were then examined under a Nikon SMZ80 microscope and identified following the keys of Bouček and Wiebes [30,31]. We then selected randomly 30 female pollinator specimens per tree for initial *Wolbachia *screening.

### DNA extraction

Each specimen was washed in MILLI-Q water before total genomic DNA was extracted non-destructively from the whole specimen using Easypure genomic DNA Extraction kits (TransGen, Beijing, China). Extracted DNA solution was used for PCR amplification and then the specimens were restored in 95% ethanol as vouchers in Institute of Zoology, Chinese Academy of Sciences.

### PCR amplification

The DNA qualities of extracts were first checked using *COI *PCR amplification (details below). The positives were then screened for *Wolbachia *infection by PCR, employing the primers *wsp*81F (5'-TGGTCCAATAAGTGATGAAGAAAC-3') and *wsp*691R (5'-AAAAATTAAACGCTACTCCA-3') [40], which amplify part of the *Wolbachia *surface protein gene (*wsp*). We also amplified the *ftsZ *and *16S rDNA *regions using primers *ftsZ *F (5'-TACTGACTGTTGGAGTTGTAACTAACGCGT-3')-*ftsZ *R (5'-TGCCAGTTGCAAGAACAGAAACTCTAACTC-3') [41] and 16SwolF (5'-TTGTAGCCTGCTATGGTATAACT-3')-16SwolR (5'-GAATAGGTATGATTTTCATGT-3') [42] to verify *Wolbachia *infection status. After initial screening, we chose five infected individuals and five uninfected ones (or less than five if unavailable) from each sample location to amplify *COI *and ITS2 fragments.

We amplified the mitochondrial *COI *fragment using the barcode primer set of LCO1490 (5'-GGTCAACAAATCATAAAGATATTGG-3') and HCO2198 (5'-TAAACTTCAGGGTGACCAAAAAATCA-3') [43,44] with the following conditions: 5 min at 94°C, followed by 35 cycles of 30 s at 94°C, 45 s at 50°C, 1 min at 72°C, and a final elongation step of 10 min at 72°C. All PCR amplifications were performed using an Eppendorf Mastercycler gradient machine (Eppendorf) in 25 μl reaction volume: Tris-HCl 20 mM (pH 8.4), KCl 20 mM, (NH_4_)_2_SO_4 _10 mM, 5 pMol of each primer, 20 mM dNTPs, 1 μl of genomic DNA (~15 ng), and 2.5 unit of TransTaq polymerase (TransGen, Beijing, China).

5 μl of each PCR product was electrophoresed through 1% agarose gel to determine the size of fragment. Positive products were then purified with EasyPure PCR purification Kit (TransGen, Beijing). Because direct sequencing of the *COI *amplicon is difficult in Hymenoptera due to a poly-T region, the purified PCR products were cloned and 2-3 positive clones were sequenced with primer M13 on an ABI3730 capillary autosequencer.

The primers ITS2F (5'-ATTCCCGGACCACGCCTGGCTGA-3') and ITS2R (5'-TCCTCCGCTTATTGATATGC-3') [45] were used to amplify ITS2. The amplification was performed with the following conditions: 5 min at 94°C, followed by 35 cycles of 30 s at 94°C, 30 s at 55°C, 30 s at 72°C, and a final elongation step of 10 min at 72°C. The ITS2 products were purified and directly sequenced.

We also sequenced the *wsp *fragments directly from purified PCR products. If the direct sequencing failed (multiple peaks or frameshifts in electropherogram profiles may reveal the presence of more than one strain of *Wolbachia*), we cloned them and sequenced 5-6 positive clones.

### Sequence analysis

The sequences were assembled and aligned in MEGA version 4.0 [46]. We then built neighbor-joining (NJ) tree for each gene region using Kimura 2-parameter (K2P) distance model and pairwise deletion in MEGA 4.0. Genetic distances between all specimens pairs were also calculated using the K2P model.

*COI *sequence polymorphism was calculated using DnaSP version 5.00.04 [47]. Polymorphism parameters estimated included nucleotide diversity (Pi), haplotype diversity (Hd), and the number of segregating sites (S). Neutrality tests were performed on the *COI *gene to determine if the frequency spectrum of polymorphisms conformed to the predictions of the neutral model of molecular evolution. Under selective neutrality, Tajima's D, Fu and Li's D and Fu and Li's F are all expected to equal zero. A value significantly less than zero indicates a higher-than-expected number of low-frequency variants, which can be caused by a recent selective sweep or population expansion or selection against weakly deleterious mutations [26,36-38], whereas a value significantly more than zero signifies low levels of both low and high frequency polymorphisms, indicating a decrease in population size or balancing selection [48].

All the sequences representing each *COI *and ITS2 haplotype and each *Wolbachia *strain have been deposited in GenBank [GenBank accession numbers HM582244-HM582279].

## Authors' contributions

XJS and DWH conceived of the study and drafted the manuscript. XJS carried out the molecular genetic analyses. JHX participated in the design of the study and helped to draft the manuscript. JMC helped to draft the manuscript. GF participated in specimen identification. All authors read and approved the final manuscript.

## Supplementary Material

Additional file 1 ***E. verticillata *NJ-trees**. a) the tree based on *COI *gene. b) the tree based on ITS2. Click here for file

Additional file 2 **Distribution of *E. verticillata***. In the pie chart, white circle represents *E. verticillata*-1, grey represents *E. verticillata*-2 and black represents *E. verticillata*-3. Click here for file

Additional file 3 **Fit of equilibrium distributions for *E. verticillata-*1 population and *E. verticillata*-2 population**. a) *E. verticillata*-1. b) *E. verticillata*-2. X axis: Pairwise Differences. Y axis: Frequency. The circles show the observed distribution of pairwise difference. The solid lines represent the expected equilibruim distributions. In equilibrium populations, the expected curves are free of waves. The observed curves with many peaks or resemblance to expected curves mean equilibrium population. On the contrary, unimodal curves represent population expansion [39]. Click here for file
