# Supplementary figures and images for: Comparisons of host mitochondrial, nuclear and endosymbiont bacterial genes reveal cryptic fig wasp species and the effects of Wolbachia on host mtDNA evolution and diversity
Source: BMC Evol Biol. 2011 Apr 1;11:86. doi: 10.1186/1471-2148-11-86 (PMC3083349; doi:10.1186/1471-2148-11-86)

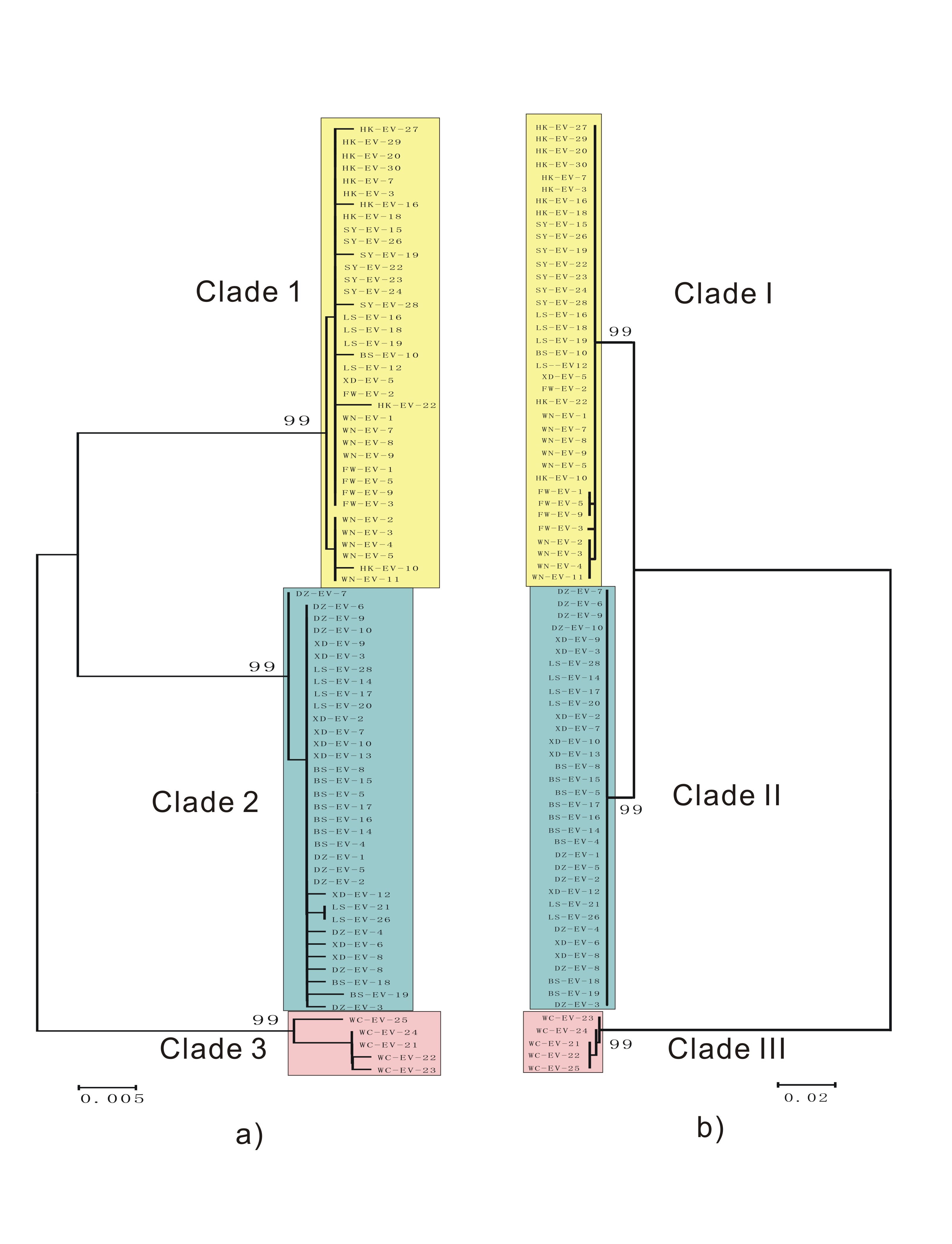

Supplement: Additional file 1 — E. verticillata NJ-trees. a) the tree based on COI gene. b) the tree based on ITS2. [file 1471-2148-11-86-S1.JPEG]

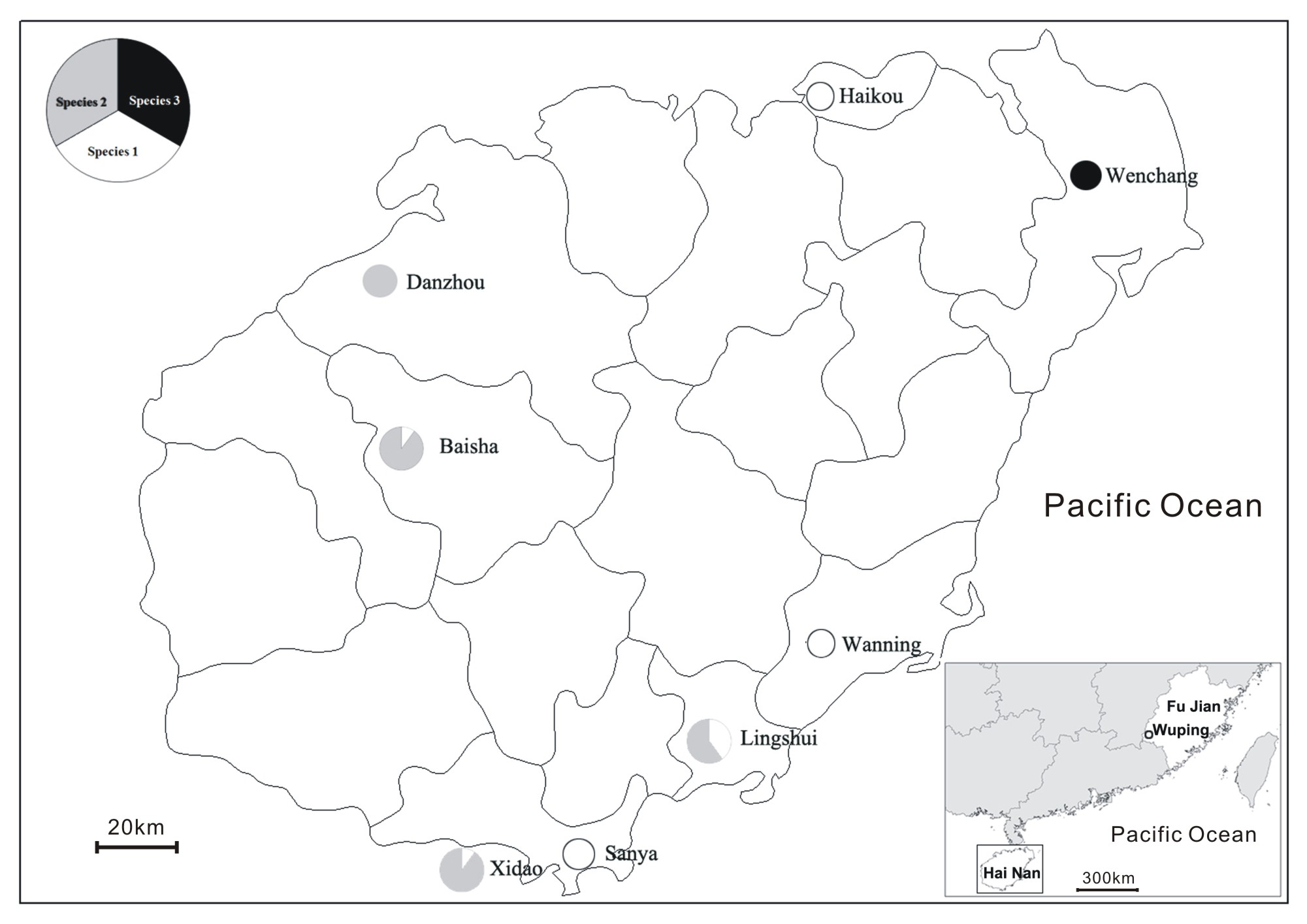

Supplement: Additional file 2 — Distribution of E. verticillata. In the pie chart, white circle represents E. verticillata-1, grey represents E. verticillata-2 and black represents E. verticillata-3. [file 1471-2148-11-86-S2.JPEG]

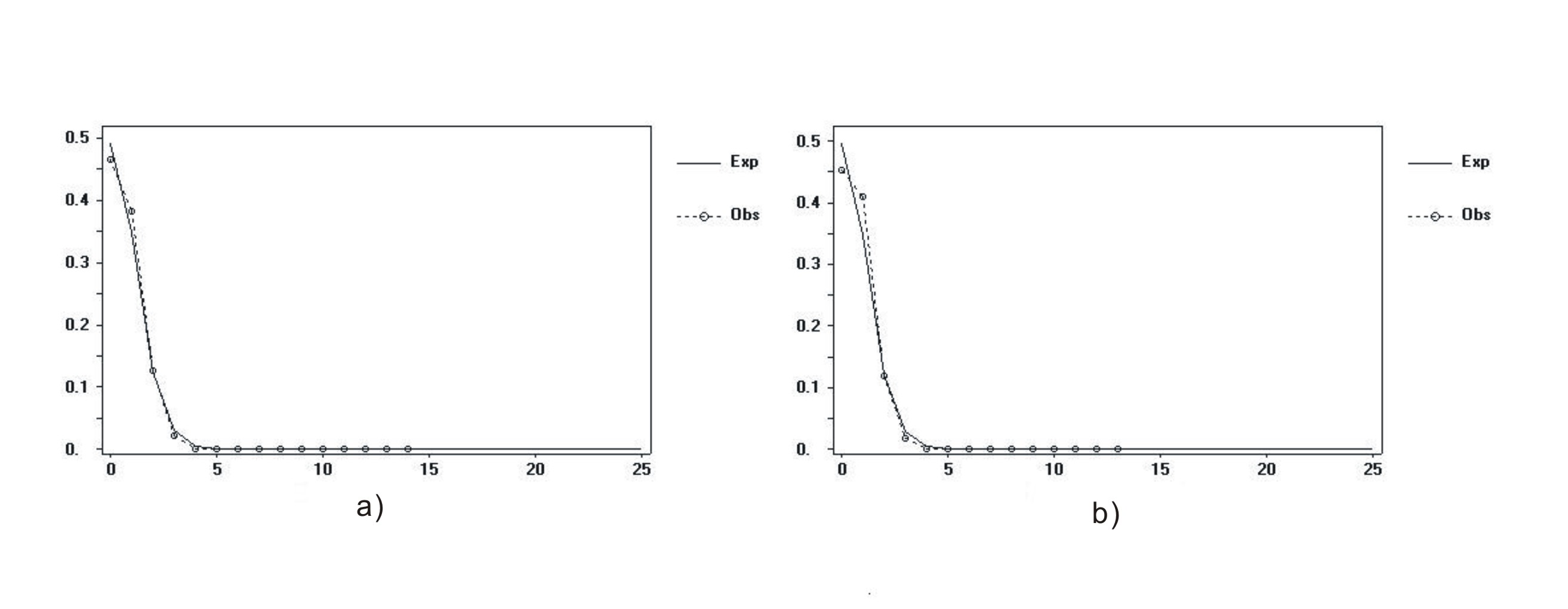

Supplement: Additional file 3 — Fit of equilibrium distributions for E. verticillata-1 population and E. verticillata-2 population. a) E. verticillata-1. b) E. verticillata-2. X axis: Pairwise Differences. Y axis: Frequency. The circles show the observed distribution of pairwise difference. The solid lines represent the expected equilibruim distributions. In equilibrium populations, the expected curves are free of waves. The observed curves with many peaks or resemblance to expected curves mean equilibrium population. On the contrary, unimodal curves represent population expansion [39]. [file 1471-2148-11-86-S3.JPEG]
